# Structure-activity relationship studies for inhibitors for vancomycin-resistant *Enterococcus* and human carbonic anhydrases

**DOI:** 10.1080/14756366.2022.2092729

**Published:** 2022-06-27

**Authors:** Weiwei An, Katrina J. Holly, Alessio Nocentini, Ryan D. Imhoff, Chad. S. Hewitt, Nader S. Abutaleb, Xufeng Cao, Mohamed N. Seleem, Claudiu T. Supuran, Daniel P. Flaherty

**Affiliations:** a Department of Medicinal Chemistry and Molecular Pharmacology, College of Pharmacy, Purdue University, West Lafayette, IN, USA; b Department of NEUROFARBA, Section of Pharmaceutical and Nutraceutical Sciences, University of Florence, Firenze, Italy; c Department of Biomedical Sciences and Pathobiology, Virginia-Maryland College of Veterinary Medicine, Virginia Polytechnic Institute and State University, Blacksburg, VA, USA; d Center for Emerging, Zoonotic and Arthropod-Borne Pathogens, Virginia Polytechnic Institute and State University, Blacksburg, VA, USA; e Purdue Institute for Drug Discovery, West Lafayette, IN, USA; f Purdue Institute of Inflammation, Immunology and Infectious Disease, West Lafayette, IN, USA

**Keywords:** Carbonic anhydrase inhibitors, vancomycin-resistant *Enterococcus*, antibiotics, drug repurposing

## Abstract

Vancomycin-resistant enterococci (VRE), consisting of pathogenic *Enterococcus faecalis and E. faecium*, is a leading cause of hospital-acquired infections (HAIs). We recently repurposed the FDA-approved human carbonic anhydrase (CA) inhibitor acetazolamide (**AZM**) against VRE agent with the likely mechanism of action for the molecules being inhibition of one, or both, of the bacterial CA isoforms expressed in VRE. To elucidate how inhibitor binding to the enzymes relates to MIC, we further characterised the inhibition constants (*K*_i_) against the *E. faecium* α-CA (*Efα*-CA) and γ-CA (*Ef*γ-CA), as well as against human CA I (hCAI) and human CA II (hCAII) to assess selectivity. We have also utilised homology modelling and molecular dynamics (MD) simulations to gain a better understanding of the potential interactions the molecules are making with the targets. In this paper, we elaborate on the SAR for the **AZM** analogs as it pertains to MIC and *K*_i_ for each CA.

## 1. Introduction

Vancomycin-resistant enterococci (VRE) is a member of the notorious group of drug-resistant ESKAPE pathogens^1^ and is considered a serious threat to public health by the Centres for Disease Control and Prevention^2^. VRE encompasses a host of *Enterococcus* species, facultative anaerobic Gram-positive bacteria that are able to withstand harsh conditions and colonise surfaces in healthcare settings^3^. The earliest report of VRE from 1899 revealed it as a leading cause of infective endocarditis^4^. Later studies also indicated that VRE is a leading cause of pelvic, neonatal and urinary tract infections (UTIs)^5^. In the 1970s, VRE primarily consisted of *Enterococcus faecalis*, which accounted for over 90% of clinical enterococcal isolates during the first wave of VRE infections^6^. However, since the 1990s, the leading causative agent of VRE has been *Enterococcus faecium* with now more than 70% of isolated strains being resistant to vancomycin^6^. Furthermore, VRE was responsible for a 10% mortality rate among nearly 55,000 reported cases in 2017^2^^,^^7^. However, the mortality rate climbs for those with systemic blood-stream infections reaching as high as 30%^8^. Patients at high risk for VRE infection include those in long-term healthcare facilities, intensive care unit patients, organ transplant patients and patients with weakened immune systems.

Lack of effective treatment is a primary reason making VRE difficult to treat. Linezolid and daptomycin have been approved by the FDA as therapeutics for treatment of systemic VRE, with daptomycin showing a slightly increased survival rate compared to linezolid^8^. However, linezolid presents toxicity concerns including myelosuppression, serotonin syndrome, neuropathy, and lactic acidosis which may limit the length of treatment^9^. The synergistic 30:70 quinupristin-dalfopristin combination was also approved by the FDA in 1998, but the combination’s high toxicity prevents the therapy from being frequently utilised, except as a last-resort measure^9^. Thus there is an urgent need for the development of new anti-VRE therapeutics.

Our team previously screened a library of FDA-approved drugs against VRE strains and discovered that the human carbonic anhydrase (hCA) inhibitor acetazolamide (**AZM**) possessed anti-enterococcal activity with a minimum inhibitory concentration (MIC) of 0.5 μg/mL^10^. Our team then carried out a hit-to-lead optimisation campaign centred on the **AZM** scaffold and generated a series of **AZM** analogs, many of which displayed significant potency against VRE strains tested^11^. However, the mechanisms of action were not fully understood. It was speculated that the molecules may be targeting the *E. faecium α*- and/or γ-carbonic anhydrases (*Efα-*CA and *Efγ*-CA, respectively) inside the bacteria.

Carbonic anhydrases (CAs) are ubiquitous metalloenzymes that exist in prokaryotes and eukaryotes. The role of CAs is to catalyse the conversion of carbon dioxide to bicarbonate, most often mediated by a Zn^2+^ ion, in order to maintain the pH homeostasis of the biological system. There are four main CA families that are genetically independent from each other: α-, β-, γ-, and δ-CAs^12^, while new families are still being discovered^13^. The α-CAs are widely distributed among vertebrates, bacteria, algae, and plants and is the only isoform expressed in humans, while β-, δ- and γ-CAs are distributed across different kingdoms, including in archaea and bacteria^14^^,^^15^. It is yet to be established whether either *Efα*-CA and/or *Efγ*-CA are essential in VRE, but CAs have been demonstrated to be essential in other pathogens such as *Helicobacter pylori*^16^^,^^17^ and *Neisseria gonorrhoeae*^18^^,^^19^. We hypothesise based on the efficacy of our known human CA inhibitor scaffold that one or both of these genes may be essential for VRE growth as well. However, the inhibitory activity of these molecules against either *Efα*-CA or *Efγ*-CA has never been characterised. Therefore, our group set out to fill this gap in knowledge and to quantify the inhibitory activity of the reported anti-VRE compounds against both VRE-CA isoforms. We then assessed the structure-activity-relationship (SAR) for the series against both VRE-CAs, compared to the activity against human isoforms hCA I and hCA II, and also investigated possible protein-ligand interactions in *Efα*-CA that could explain binding affinity using molecular dynamics (MD) simulations. The results from these studies are reported herein.

## 2. Materials and methods

### 2.1. Expression and purification of VRE CAs

Both *Efα-*CA and *Efγ*-CA were recombinantly expressed and purified as follows. A pHis2 plasmid (GenScript) containing either the *Ef*α-CA sequence or *Efγ*-CA sequence was used for the expression of hexa-histidine tagged protein in a bacterial culture. The plasmid was transformed into competent BL21 (DE3) *E. coli* cells (Novagen, catalog no. 70953) according to manufacturer recommendation. Starter cultures were grown at 37 °C with shaking at 225 rpm overnight. One litre of autoclaved Luria-Bertani broth containing 100 µg/mL ampicillin and 1 mM ZiCl_2_ was inoculated with a 10-ml aliquot of the starter culture and grown at 37 °C with shaking at 225 rpm to an OD of 0.8 before being cold shocked and induced with 500 µL of IPTG (1 M). The induced cultures were grown for 16 h at 17 °C with shaking at 225 rpm. Bacterial cell pellets were spun down at 4000 *g* for 20 min and resuspended in 1× PBS containing 0.5 mM TCEP, 5% glycerol, pH = 7.4 to which 5 mg lysozyme was added to aid in lysis. The resuspended bacterial cells were incubated on ice for 20 min and then lysed via sonication. Lysed bacterial cells were pelleted by centrifugation at 14,000 *g* for 1 h to remove cellular debris, and the supernatant was loaded onto a nickel-NTA column equilibrated with 1x PBS containing 0.5 mM TCEP, 5% glycerol, pH = 7.4. Once flowthrough was collected, the protein was eluted using a 0–500 mM imidazole stepwise gradient in the same equilibration buffer, and fractions were collected. Fractions that contained the desired protein, as determined by SDS-PAGE, were combined and subjected to TEV protease for His-tag cleavage. After the addition of TEV, the protein was dialysed for 4 h against 1× PBS containing 0.5 mM TCEP, 5% glycerol, pH = 7.4. The dialysed protein solution was loaded back onto a nickel-NTA column equilibrated with 1x PBS containing 0.5 mM TCEP, 5% glycerol, pH = 7.4 for the subtraction of TEV followed by its elution with the same buffer containing 500 mM imidazole. The protein eluted prior to the imidazole was concentrated using Amicon Ultra Centrifugal Filters and purified by size-exclusion chromatography (SEC) on an S100 column using running buffer 1x PBS containing 0.5 mM TCEP, 5% glycerol, pH = 7.4. Fractions that contained the protein of interest as confirmed by SDS-PAGE were concentrated, flash frozen, and placed in −80 °C for future use.

### 2.2. Carbonic anhydrase CO_2_ hydration catalytic assay and K_i_ determination

The assay was performed according to previously published protocols^19–24^. Recombinant *Efα*-CA and *Efγ*-CA was obtained as described above and hCA I and hCA II were purchased from Millipore Sigma (hCA I Catalog# C4396-5MG; hCA II Catalog #C6624-500UG). *K*_i_ values were determined from inputting the IC_50_ values into the Cheng–Prusoff equation^25^ for *K*_i_ from catalytic inhibition constants.

### 2.3. Protein preparation and ligand docking

All computational protein and ligand preparation and docking was performed using programs available within the Maestro interface of the Schrödinger Small Molecule Drug Discovery Suite (Schrödinger, LLC, New York, NY, software release 2021–2). The *Efα*-CA homology model built from previous work^13^ was utilised for protein preparation. The homology model was processed using the Protein Preparation Wizard from the Schrödinger platform. During the pre-processing step, bond orders were assigned, the CCD database was used, hydrogens were added, zero-order bonds to metals were created, disulphide bonds were created, and heteroatom states of pH 7.0 ± 2.0 were generated with Epik. The structure was then refined by sampling water orientations using PROPKA pH 7.4, followed by the removal of waters 3.0 Å beyond heteroatoms and with fewer than 3 hydrogen bonds to non-waters. The last step of refinement involved restrained minimisation by converging heavy atoms to an RMSD of 0.3 Å using the OPLS4 forcefield.

To prepare for ligand docking, **AZM** was placed in the known catalytic binding site near the Zn^2+^ ion. A grid was generated with the Receptor Gride Generation program in Glide using **AZM** as the workspace reference ligand and setting the Zn^2+^ ion as a metal coordination constraint. All other parameters were set to default settings. Ligands were prepared using LigPrep with default ligand preparation settings. After ligand preparation, **AZM** and the ligands of interest were docked into the binding site with Glide using Extra Precision (XP) and default docking settings. The ligand poses with the best Glide-XP scores that formed a coordination between the sulphonamide moiety and the Zn^2+^ ion were carried forward into the molecular dynamic simulations.

### 2.4. Molecular dynamics simulations of ligand binding

All MD simulations were performed using the Desmond (D. E. Shaw Research) program available in the Maestro interface of the Schrodinger platform (software release 2021–2). In preparation for the MD simulations, a solvation model was built with System Builder powered by Desmond using the following parameters. The solvent model was predefined as TIP3P with boundary conditions set to orthorhombic. The box size calculation was set to buffer with box volume minimised. Ion and salt placement was excluded within 5 Å of the ligand, and ions were placed to neutralise the calculated charge. Additionally, 0.15 M NaCl was added to the model.

Once the solvation model was built, molecular dynamics simulations were run using Desmond. Each simulation time was set to 72 ns with recording intervals of 10 ps. Ensemble class was set to NPT with a temperature of 300.0 K and pressure of 1.01325 bar. The remaining simulation parameters were set to default settings. Convergence time for each ligand was determined by noting the time at which the reported ligand RMSD appeared to have stabilised.

## 3. Results and discussion

### 3.1. Structure-activity relationship data

To further understand the structure-activity relationship, we have collected the inhibition constants (*K*_i_s) against *Efα*-CA, *Efγ*-CA, hCA I and hCA II (Table 1) for all the analogs developed against VRE reported in our previous studies^11^

**Table 1.** Inhibition constants (*K*_i_) against the α-EfCA, γ-EfCA, hCAI and hCAII.

Cmpd	Structures	*K*_i_ (nM)			
		*Efα*-CA	*Efγ*-CA	hCA I^a^	hCA II^a^
**AZM**	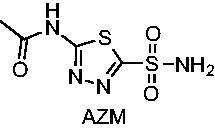	56.7	322.8	250	12.5
**1**	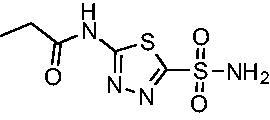	37.5	250.1	235.4	37.2
**2**	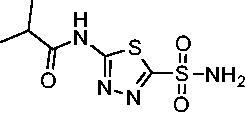	23.7	218.4	179.7	30.9
**3**	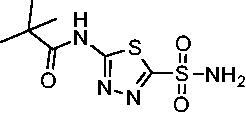	29.8	440.8	167.3	9.5
**4**	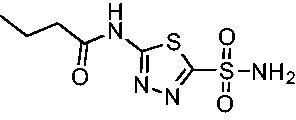	50.1	325.3	212.5	22.3
**5**	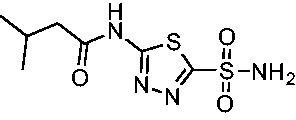	62.7	279	215.1	55.6
**6**	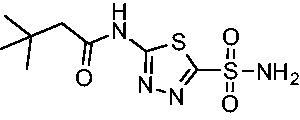	69.6	389.5	230.9	7.3
**7**	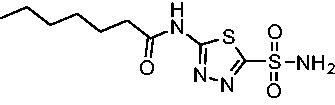	78.8	631.7	327.5	22.7
**8**	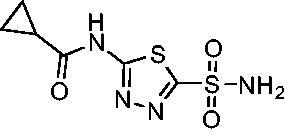	36.2	192.7	156.9	24.6
**9**	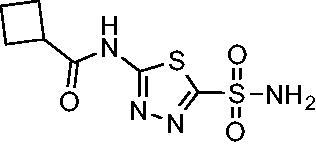	27.2	285.9	125.2	41.5
**10**	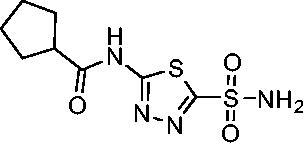	9.8	194.9	76.5	26.2
**11**	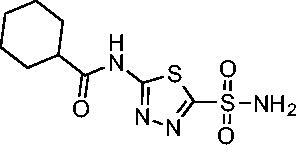	49.3	131.1	63.3	20.2
**12**	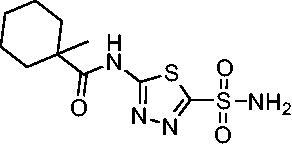	44.5	239.6	117.3	47.7
**13**	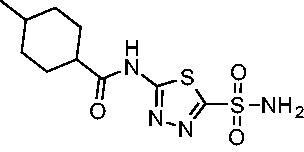	29.6	345.3	151.9	26
**14**	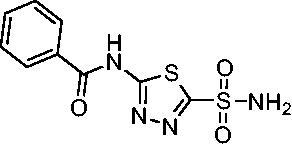	56.2	94.8	108.6	29
**15**	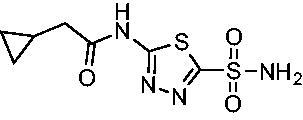	11.7	310.5	372.4	7.6
**16**	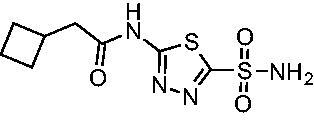	18.4	408.4	77.8	4.9
**17**	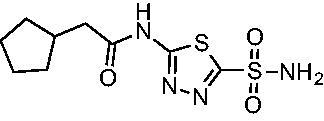	21.9	232.8	86.3	19.4
**18**	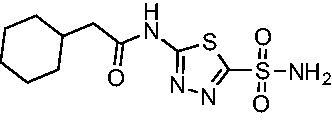	14.5	304.8	58.7	24.5
**19**	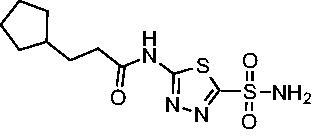	39.3	244.6	190.2	10.2
**20**	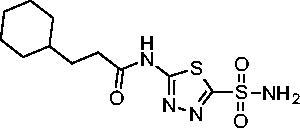	66.9	345.9	945.9	0.32
**21**	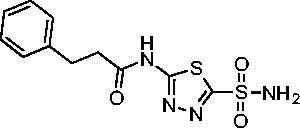	6.4	148.4	855.3	8.1
**22**	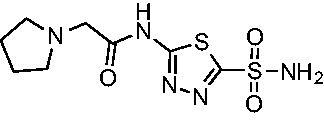	38.1	92.8	64.7	58.1
**23**	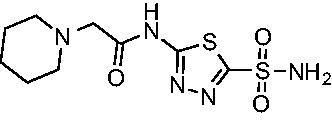	29.5	110.6	53.4	20.9
**24**	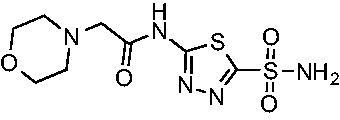	20.1	56.4	14	0.9
**25**	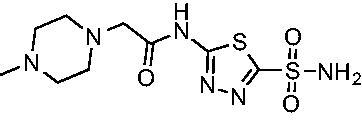	81.5	64.7	9.6	1.6
**26**	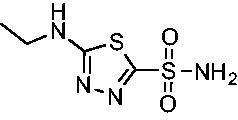	165.2	504.5	701	47.2
**27**	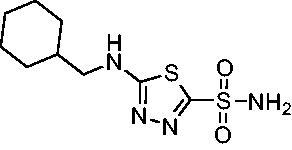	117.3	398	1135	78.4
**28**	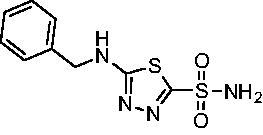	163.5	282	1623	103.8
**29**	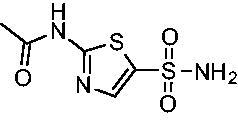	374.7	1129	465.2	97.2
**30**	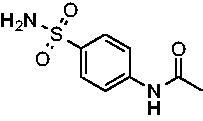	179.7	528.6	1331	67.7

^a^Ki data for analogs against hCA isoforms previously reported^21^.

Compared with **AZM**, the addition of branched alkyl bulk adjacent to the amide carbonyl position generally improved potency against both *Efα*-CA and *Efγ*-CA with one exception. Progression from methyl (**AZM**) to ethyl (**1**) to *iso-*propyl (**2**) provided stepwise improvement against *Efα*-CA with *K*_i_ values of 56.7, 37.5 and 23.7 nM, respectively. A similar trend was observed against *Efγ*-CA, as **AZM** displayed a *K*_i_ of 322.8 nM and was improved to 218.4 nM in the *iso*-propyl derivative **2**. Interestingly, the *tert*-butyl analog **3** maintained similar potency against *Efα*-CA (*K*_i_ = 29.8 nM) compared to **2** but led to about a 2-fold reduction of potency against *Efγ*-CA (*K*_i_ = 440.8 nM) compared to **2**. When the alkyl group was linearly extended an additional methylene from the amide carbonyl, the series of analogs with increased alkyl branching (**4−6)** was generally less potent against both EfCAs compared to the series with the branch point adjacent to the carbonyl. Conversely, extending the linear alkyl chain to an *n*-hexyl substituent in analog **7** increased the *K*_i_ values against *Efα*-CA and *Efγ*-CA to 78.8 nM and 631.7 nM, respectively.

A set of cycloalkyl derivatives provided additional, tractable SAR data points against both *Efα*-CA and *Efγ*-CA. The first cohort of matched molecular pairs in which the cycloalkane branch point was directly adjacent to the amide carbonyl followed a similar trend against *Efα*-CA as was observed for the branched alkanes. For example, expansion of the ring from three to four to five carbons improved potency for the series with the cyclopentane analog **10** displaying the most potent *K*_i_ (9.8 nM) of any analog thus far. However, similarly to what was observed for the branched alkanes, there appears to be a potency limit with respect to increased ring size as the cyclohexane derivative **11** was the least potent of this group (*K*_i_=49.3 nM), followed by the quaternary 1-methyl substituted **12** (*K*_i_=44.5 nM). Placing the methyl substituent at the 4-position in **13** slightly improved *Efα*-CA binding (*K*_i_=29.6 nM) but not to the level observed for the cyclopentane derivative **10**. Interestingly, *Efγ*-CA preferred the cycloalkane modifications. Contrary to the *Efα*-CA binding data, the cyclohexane derivative **11** was the most potent of the series with a *K*_i_ of 131.1 nM. Increasing the alkyl branching on the cyclohexane ring at either the 1- or the 4-position as in analogs **12** and **13**, respectively, reduced *Efγ*-CA binding to *K*_i_ values over 200 nM.

The second set of cycloalkane derivatives (**15−18**), in which a methylene linker is inserted between the carbonyl and the ring generally improved potency against *Efα*-CA across the board with all analog *K*_i_s ranging from 11−22 nM. The cyclopentane analog **17** did display about 2-fold reduced potency compared to its matched molecular paired analog **10**, but this cohort generally was the best performing as a group against *Efα*-CA. However, just as observed for the branched alkane analogs (**4−7**), extension of the linker was detrimental to *Efγ*-CA activity for analogs **15−18** with values consistently less potent compared to the nearest neighbour counterparts not containing the methylene insertion. Adding a second methylene to the linker for the cyclopentane (**19**) and cyclohexane (**20**) derivatives reduced potency against both isoforms compared to the single methylene derivatives.

Two sets of matched molecular pairs compare the saturated cyclohexane (**11** and **20**) to a phenyl substitution (**14** and **21**) with varying linker length from the carbonyl. For the cyclohexane to phenyl substitution attached directly to the carbonyl (**11** and **14**) the activity was essentially equipotent against *Efα*-CA, while the phenyl derivative was more active against *Efγ*-CA (*Efα*-CA *K*_i_ = 131.1 nM, *Efγ*-CA *K*_i_ = 94.8 nM). This activity difference between isoforms was more pronounced with respect to the matched molecular pair containing a two-methylene linker (**20** and **21**). In this case, the phenyl-containing derivative was superior to the cyclohexane against both EfCAs, with a 10-fold increase in activity against *Efα*-CA with a *K*_i_ value of 6.4 nM and a 2-fold improvement against *Efγ*-CA with a *K*_i_ value of 148.4 nM. One takeaway from this data is that an aromatic pendant group seems to be preferred for *Efγ*-CA activity, but only in some instances for *Efα*-CA activity.

Another general trend that was observed for EfCA activity involves the polar pendant group in analogs **22−25**. Addition of heteroatoms into the cycloalkane ring system were generally tolerated for *Efα*-CA with *K*_i_ values ranging from approximately 20−80 nM with the least potent being the *N*-methylpiperazine analog **25** and most potent being the morpholine derivative **24**. However, this cohort consistently outperformed all other analogs with respect to *Efγ*-CA with *K*_i_ values ranging from 56−110 nM suggesting a preference for polarity in the active site of *Efγ*-CA. Analog **24** was the most potent in this series against both *Efα*-CA and *Efγ*-CA with *K*_i_ values of 20.1 nM and 56.4 nM, respectively.

The final set of analogs were designed with targeted modifications to remove different atoms in the scaffold to determine their essentiality for binding the EfCAs. In analogs **26−28,** the amide carbonyl was removed, leaving three analogs with simply an amine linkage. This resulted in a reduction of binding affinity by more than 2- to 3-fold for both *Efα*-CA and *Efγ*-CA across the set when compared to the carbonyl-containing counterparts (**AZM**, **11**, and **14**, respectively). Modification of the thiadiazole central ring to the thiazole by removing the nitrogen directly adjacent to the sulphonamide in **29** resulted in a 6-fold reduction in binding affinity against *Efα*-CA and a 3.5-fold reduction against *Efγ*-CA, indicating that this particular nitrogen is important for binding against both isoforms. Modification of the central thiadiazole to the benzenesulfonamide in **30** also resulted in reduction of binding and antimicrobial activity against both isoforms.

In general, modifications that improved activity against the *Efα*-CA also improved activity against the human isoform hCA II (an α-CA isoform), while improving selectivity over hCA I. For example, the extension of the alkyl linker to two methylene units improved hCA II activity but was detrimental to hCA I activity, while maintaining potency against *Efα*-CA. Analog **21** with the phenyl moiety was among the most potent against *Efα*-CA with the widest selectivity window over hCA I of more than 100-fold. The data also suggest the polar pendant groups were well tolerated among all CAs tested with the morpholine derivative providing sub-56 nM *K*_i_ values across all four CAs tested.

Another point to note is that no clear correlative trends were observed between EfCA *K*_i_ values and MIC potency, at least when considering the most potent antibacterial analogs **6**, **7**, and **20** as these analogs (MIC values of 0.015, 0.015, and 0.007 µg/mL, respectively, full data set published by Kaur et al.^11^) generally lagged behind the rest in terms of EfCA binding. Alternatively, for the molecules that displayed significantly reduced antibacterial activity (**26−30**) there were tangible reductions in EfCA activity that could, at least in part, explain the reduced antibacterial activity. However, additional variables may confound the comparisons between EfCA and antimicrobial activity such as permeability of the molecules to reach the EfCA within the bacterial cell, differential essentiality between the two EfCAs for bacterial survival, or the possibility of additional targets. At this point more information is necessary to draw clear correlative conclusions about the relationship between EfCA inhibition and antibacterial activity.

### 3.2. MD simulation

**Figure 1.**
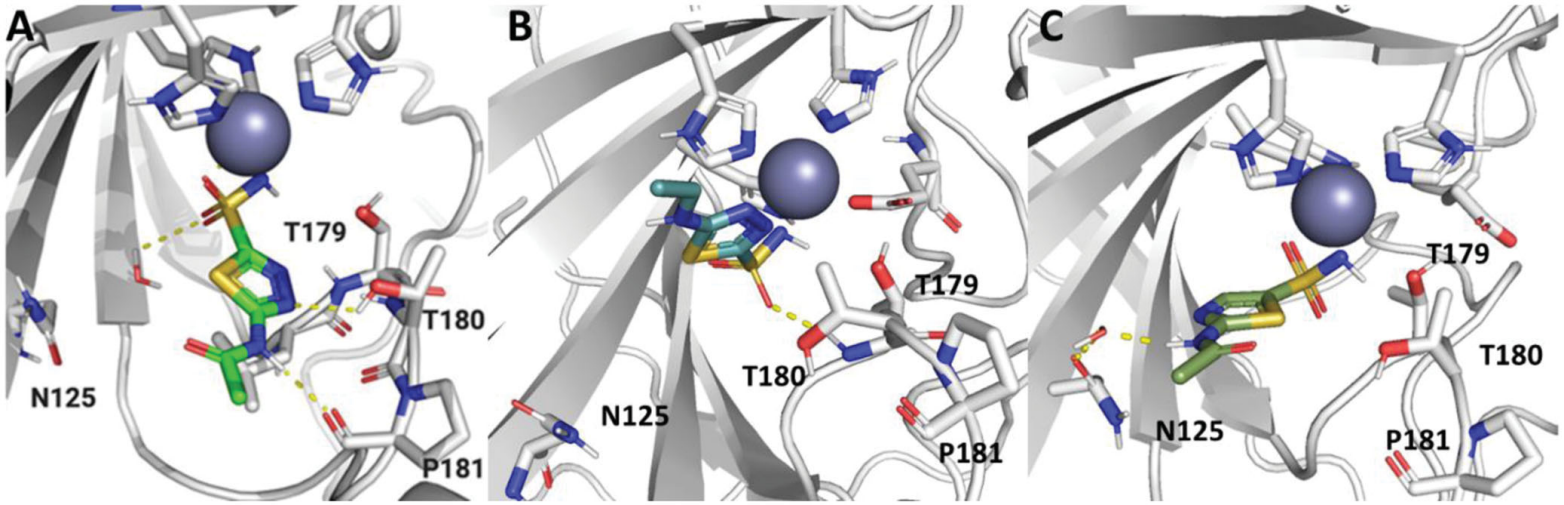
Ligand poses for **AZM**^11^, **26,** and **29** were generated by MD simulation in the active site of the *Efα*-CA at convergences of 10 ns, 41 ns, and 63 ns, respectively. Ligands, residues, and waters important for ligand interactions are shown as sticks. Polar hydrogens are shown for clarity of proposed hydrogen bond interactions (yellow dashed lines). Homology model of *Efα*-CA (gray ribbons) was used as the model. Catalytic Zn^2+^ is shown as a dark blue sphere. (A) Generated pose for **AZM** (green sticks) in the *Efα*-CA site^11^. (B) Generated pose for **26** (teal sticks) in the *Efα*-CA site. (C) Generated pose for **29** (olive sticks) in the *Efα*-CA site. Images were generated using PyMol.

MD simulations were performed in an attempt to elucidate binding interactions of particular analogs with *Efα*-CA that could explain their observed inhibition constants. The data shown for **AZM** and **20** was reported in previous work^11^. These MD simulations were run using the GPU-accelerated Desmond (D. E. Shaw Research) software package accessed through the Maestro interface of the Schrodinger platform (software release 2021–2). The MD simulation data further supported the experimental data that the amide bond of the acetazolamide scaffold is crucial for ligand binding to the *Efα*-CA active site. In Figure 1, the amide nitrogen of **AZM** is shown to be forming a hydrogen bond with P181 during 68% of simulation, whereas **26** lacks this hydrogen bond. This could be due to the additional rotatable bond introduced in **26**, thereby increasing the flexibility of this analog. The primary interaction present in the simulation data for **26** was a hydrogen bond formed between one sulphonamide oxygen and the T179 backbone present for 80% of the simulation time. Likewise, MD simulation data suggested that the thiadiazole nitrogen nearest to the sulphonamide moiety is necessary for ligand binding. When this nitrogen is missing in **29,** the thiadiazole core is flipped relative to its position in **AZM,** and there is an overall lack of ligand interactions with binding site residues which could explain the 7-fold increase in *K*_i_ observed in the binding data for *Efα*-CA (Table 1). The only interactions reported were water-mediated hydrogen bonds which occurred less than 30% of the duration of the simulation.

**Figure 2.**
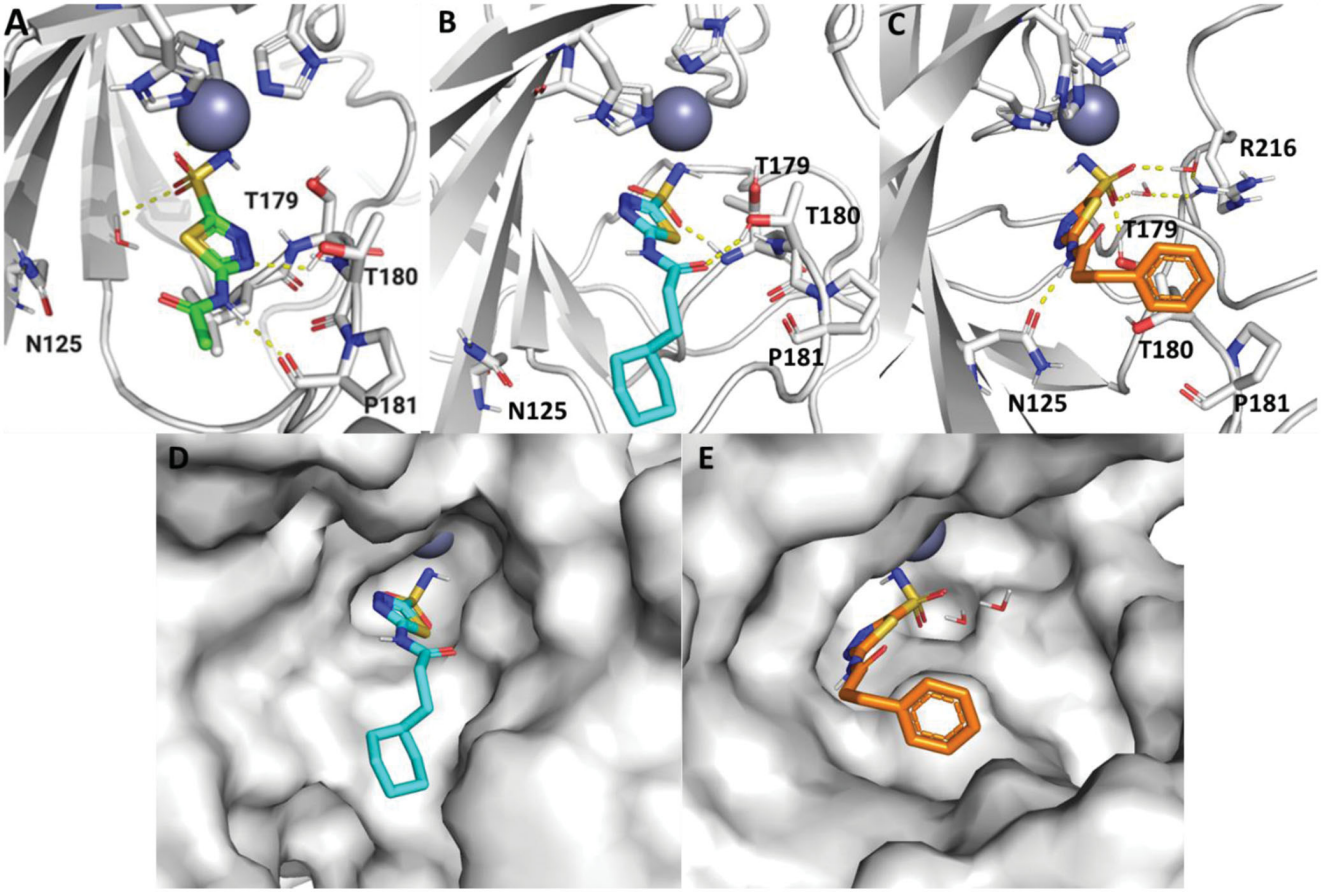
Ligand poses for **AZM**^11^, **20,** and **21** were generated by MD simulation in the active site of the *Efα*-CA at convergences of 10 ns, 10 ns, and 48 ns, respectively. Ligands, residues, and waters important for ligand interactions are shown as sticks. Polar hydrogens are shown for clarity of proposed hydrogen bond interactions (yellow dashed lines). Homology model of *Efα*-CA (gray ribbons) was used as the model. Catalytic Zn^2+^ is shown as dark blue sphere. (A) Generated pose for **AZM** (green sticks) in the Efα-CA site^11^. (B) Generated pose for **20** (cyan sticks) in the Efα-CA site. (C) Generated pose for **21** (orange sticks) in the *Efα*-CA site. (D) Generated pose for **20** (cyan sticks) with protein surface representation in the *Efα*-CA site^11^. (E) Generated pose for **21** (orange sticks) with protein surface representation in the *Efα*-CA site. Images were generated using PyMol.

MD Simulations were also utilised to analyse the differences in binding interactions between **20** and **21** to elaborate on the 10-fold difference in *K*_i_ observed in the binding assays (Table 1). In Figure 2, the relative poses of **20** and **21** at convergence are quite distinct despite only differing by the presence of aromaticity in the end of the hydrophobic moiety. The MD simulation reports revealed that the primary interaction observed for **20** was that of a hydrogen bond formed between T180 and the amide oxygen that occurred for 88% of the simulation. However, in the case of **21**, this interaction was only present 20% of the time. Instead, water molecules were present in the binding site, resulting in water-mediated hydrogen bonds between the sulphonamide and R216 as well as between the sulphonamide and E102 (not pictured due to low prevalence at convergence), all of which were observed for 24–47% of the duration of the simulation. A protein RMSF analysis revealed that there was a much higher flexibility of the T179 and T180 residues in the simulation of **21** (3.2 Å) compared to the simulation of **20** (0.6 Å)^11^. As a result of this flexibility and presence of water molecules, **21** was positioned nearer to N125, forming a hydrogen bond between the residue and the amide nitrogen of **21** for 44% of the MD simulation. Additionally, the phenyl ring in **21** was angled towards W9 (not shown) forming a hydrophobic interaction for 25% of the simulation duration, an interaction not observed in **20**. This could be due to increased protein flexibility in the N-terminus for the **21** MD simulation which was not observed in that of **20**. Why the **21** MD simulation suggested presence of water molecules in the binding site and increased residue flexibility compared to **20** when the ligand structures are highly similar remains unclear. The final observation from the MD simulation is that the predicted pose for the hydrophobic tail containing **21** indicates that the thiadiazole heterocycle is flipped in the active site by approximately 180° when compared to **AZM**. This was consistent with our previous observation for the same alternative orientiation of the thiadiazole for **20**^13^ indicating this may be a feature of this scaffold with the hydrophobic tail extended.

## 4. Conclusion

This report documents pharmacological inhibition of both *Efα*-CA and *Efγ*-CA for the first time. The **AZM**-based thiadiazole CAIs previously reported as potent anti-VRE inhibitors were screened for their ability to inhibit both *Efα*-CA and *Efγ*-CA in the CO_2_ hydration assay. It was observed that the scaffold was generally more potent against the *Efα*-CA compared to the *Efγ*-CA isoform. Increase of alkyl branching up to a tertiary alkane was generally preferred for inhibitors activity against *Efα*-CA. Linker length also played a role in *Efα*-CA inhibition with the single-methylene linker being preferred over no-linker and the di-methylene linker when comparing nearest neighbour analogs. Some SAR diverged for *Efγ*-CA. For example, the cycloalkane substituted analogs with no linker between the carbonyl and ring were among the most potent of the hydrophobic derivatives against this isoform. The polar pendant groups displayed the best combination of potency and the only analogs with consistent *K*_i_ values below 110 nM against both EfCA isoforms. Finally, targeted modifications such as removal of the carbonyl or alteration of the thiadiazole core had detrimental effects on potency against both isoforms suggesting that these moieties are beneficial for inhibition. Overall, there were no correlative trends with regard to EfCA inhibition and improved antibacterial potency towards VRE; however, molecules that exhibited highly-reduced antimicrobial activity did display 2.5- to 6-fold reduced EfCA inhibition compared to **AZM**. MD simulations further supported the essentiality of particular scaffold elements such as the amide oxygen and thiadiazole nitrogen adjacent to the sulphonamide by demonstrating an overall lack of direct binding interactions of the *Efα*-CA homology model with analogs missing these structural elememts. In summation, the data presented provides an initial assessment of SAR and the first reported inhibitory activity against *Efα*-CA and *Efγ*-CA.
